# Forming-free bipolar resistive switching in nonstoichiometric ceria films

**DOI:** 10.1186/1556-276X-9-45

**Published:** 2014-01-27

**Authors:** Muhammad Ismail, Chun-Yang Huang, Debashis Panda, Chung-Jung Hung, Tsung-Ling Tsai, Jheng-Hong Jieng, Chun-An Lin, Umesh Chand, Anwar Manzoor Rana, Ejaz Ahmed, Ijaz Talib, Muhammad Younus Nadeem, Tseung-Yuen Tseng

**Affiliations:** 1 Department of Electronics Engineering and Institute of Electronics, National Chiao Tung University, Hsinchu 30010, Taiwan; 2 Department of Materials Science and Engineering, National Chiao Tung University, Hsinchu 30010, Taiwan; 3 Department of Physics, Bahauddin Zakariya University, Multan 60800, Pakistan

**Keywords:** Resistive switching, Space charge-limited conduction (SCLC), Metal-insulator-metal structure, Cerium oxide, Oxygen vacancy

## Abstract

The mechanism of forming-free bipolar resistive switching in a Zr/CeO_*x*_/Pt device was investigated. High-resolution transmission electron microscopy and energy-dispersive spectroscopy analysis indicated the formation of a ZrO_*y*_ layer at the Zr/CeO_*x*_ interface. X-ray diffraction studies of CeO_*x*_ films revealed that they consist of nano-polycrystals embedded in a disordered lattice. The observed resistive switching was suggested to be linked with the formation and rupture of conductive filaments constituted by oxygen vacancies in the CeO_*x*_ film and in the nonstoichiometric ZrO_*y*_ interfacial layer. X-ray photoelectron spectroscopy study confirmed the presence of oxygen vacancies in both of the said regions. In the low-resistance ON state, the electrical conduction was found to be of ohmic nature, while the high-resistance OFF state was governed by trap-controlled space charge-limited mechanism. The stable resistive switching behavior and long retention times with an acceptable resistance ratio enable the device for its application in future nonvolatile resistive random access memory (RRAM).

## Background

A metal-insulator-metal (MIM) structure-based resistive random access memory (RRAM) device has attracted much attention for next-generation high-density and low-cost nonvolatile memory applications due to its long data retention, simple structure, high-density integration, low-power consumption, fast operation speed, high scalability, simple constituents, and easy integration with the standard metal oxide semiconductor (MOS) technology [1]. In addition to transition metal oxide-based RRAMs [2,3], many rare-earth metal oxides, such as Lu_2_O_3_, Yb_2_O_3_, Sm_2_O_3_, Gd_2_O_3_, Tm_2_O_3_, Er_2_O_3_, Nd_2_O_3_, Dy_2_O_3_, and CeO_2_[4-10], show very good resistive switching (RS) properties. Among them, CeO_2_ thin films having a strong ability of oxygen ion or oxygen vacancy migration attract a lot of attention for RRAM applications [8-10]. CeO_2_ is a well-known rare-earth metal oxide with a high dielectric constant (26), large bandgap (6 eV), and high refractive index (2.2 to 2.3). The cerium ion in the CeO_2_ film exhibits both +3 and +4 oxidation states, which are suitable for valency change switching process [11,12]. Forming-free resistive switching and its conduction mechanism are very important for nonvolatile memory applications. In addition, oxygen vacancies or ions play a unique role in the resistive switching phenomenon [13]. Therefore, CeO_2_ is expected to have potentials for applications in nonvolatile resistive switching memory devices [14]. However, there are quite limited reports on the resistive switching properties of CeO_2_.

Here, we report the forming-free bipolar resistive switching properties of a nonstoichiometric CeO_*x*_ film having a Zr/CeO_*x*_/Pt device structure. The effect of the Zr top electrode on the resistive switching behavior of the CeO_*x*_ film is investigated. It is expected that the Zr top electrode reacts with the CeO_*x*_ layer and forms an interfacial ZrO_*y*_ layer. This reaction may be responsible for creating a sufficient amount of oxygen vacancies required for the formation and rupture of conductive filaments for resistive switching. In this study, we have found that the CeO_*x*_-based RRAM device exhibits good switching characteristics with reliable endurance and data retention, suitable for future nonvolatile memory applications.

## Methods

A 200-nm-thick silicon dioxide (SiO_2_) layer was thermally grown on a (100)-oriented p-type Si wafer substrate. Next, a 50-nm-thick Pt bottom electrode was deposited on a 20-nm-thick Ti layer by electron beam evaporation. The 14- to 25-nm-thick CeO_*x*_ films were deposited on Pt/Ti/SiO_2_/Si at room temperature with a gas mixture of 6:18 Ar/O_2_ by radio-frequency (rf) magnetron sputtering using a ceramic CeO_2_ target. Prior to rf sputtering at 10-mTorr pressure and 100-W power, the base pressure of the chamber was achieved at 1.2 × 10^-6^ Torr. Finally, a 30-nm-thick Zr top electrode (TE) and a 20-nm-thick W TE capping layer were deposited by direct current (DC) sputtering on the CeO_*x*_ film through metal shadow masks having 150-μm diameters to form a sandwich MIM structure. The W layer was used to avoid the oxidation of the Zr electrode during testing. Structural and compositional characteristics of the CeO_*x*_ films were analyzed by X-ray diffraction (XRD; Bede D1, Bede PLC, London, UK) and X-ray photoelectron spectroscopy (XPS; ULVAC-PHI Quantera SXM, ULVAC-PHI, Inc., Kanagawa, Japan) measurements. The film thickness and interfacial reaction between Zr and CeO_*x*_ were confirmed by high-resolution cross-sectional transmission electron microscopy (HRTEM). Elemental presence of deposited layers was investigated by energy-dispersive spectroscopy (EDX). Electrical current–voltage (*I*-*V*) measurement was carried out using the Agilent B1500A (Agilent Technologies, Santa Clara, CA, USA) semiconductor analyzer characterization system at room temperature. During electrical tests, bias polarity was defined with reference to the Pt bottom electrode.

## Results and discussion

**Figure 1:**
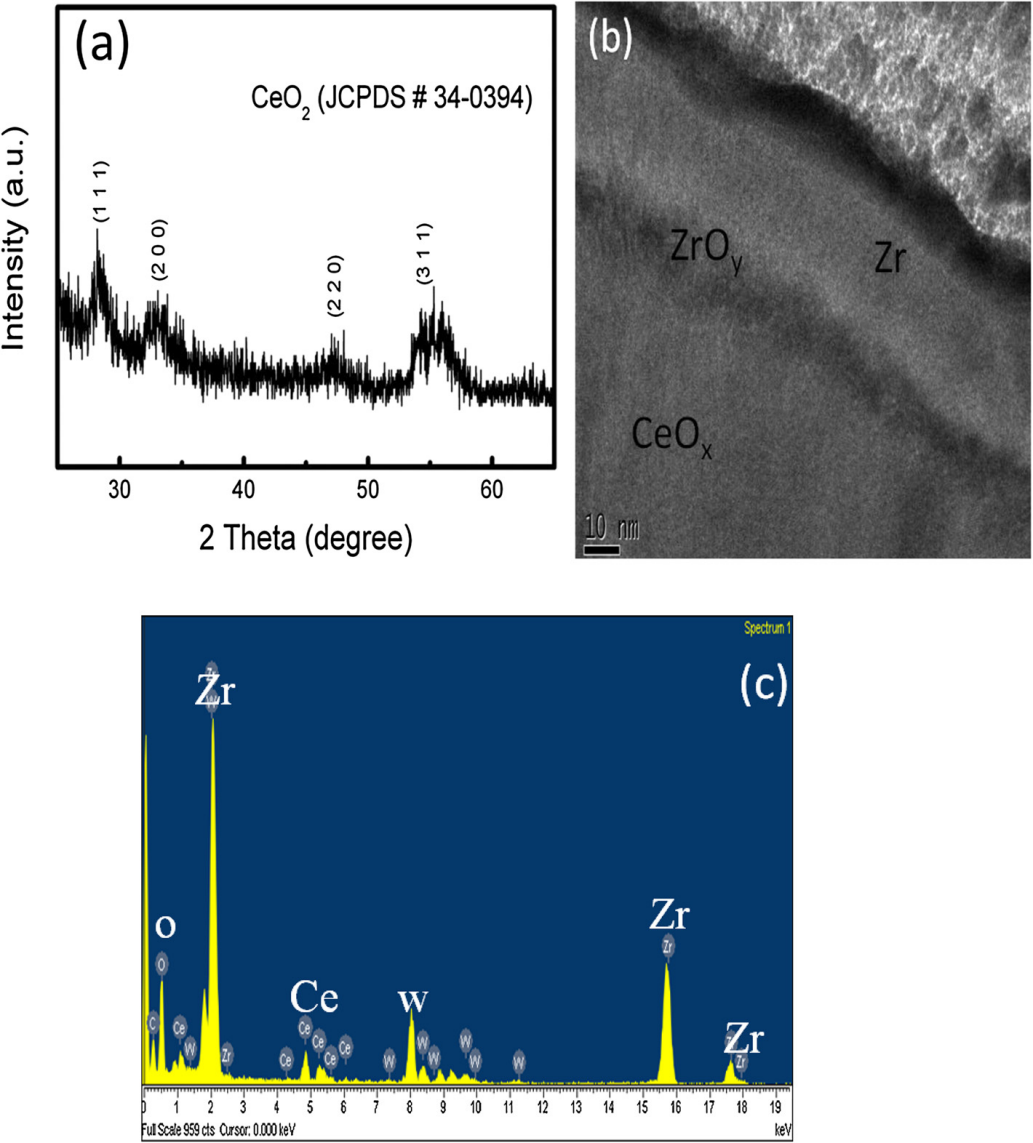
**XRD pattern of the CeO**_***x***_**film and cross-sectional TEM and EDX images of the Zr/CeO**_***x***_**/Pt device. (a)** XRD pattern of the CeO_*x*_ film deposited on Si wafer at room temperature. **(b)** Cross-sectional TEM image of the Zr/CeO_*x*_/Pt device. **(c)** EDX image of the Zr/CeO_*x*_/Pt device.

Figure 1a shows the grazing angle (3°) XRD spectra of the CeO_*x*_ thin film deposited on Si (100) substrate. It indicates that the CeO_*x*_ film possesses a polycrystalline structure having (111), (200), (220), and (311) peaks, corresponding to the fluorite cubic structure (JCPDS ref. 34–0394). From the XRD analysis, the broad and wide diffraction peaks demonstrate that the CeO_*x*_ film exhibits poor crystallization. This could be due to the small thickness (approximately 14 nm) of the film. Figure 1b shows the cross-sectional HRTEM image of the Zr/CeO_*x*_/Pt device, which indicates that the ZrO_*y*_ layer is formed between CeO_*x*_ and Zr interfaces. Figure 1c depicts the EDX spectra of the CeO_*x*_ film. The elemental composition of the Zr/CeO_*x*_/Pt was determined by energy dispersion. The results from the EDX analysis that showed the main component present in this structure were O (38.41%), Zr (34. 05%), and Ce (3.83%). An oxygen peak at about 0.52 keV and Zr peaks at about 22.5 and 15.60 keV can be observed in the spectra.

**Figure 2:**
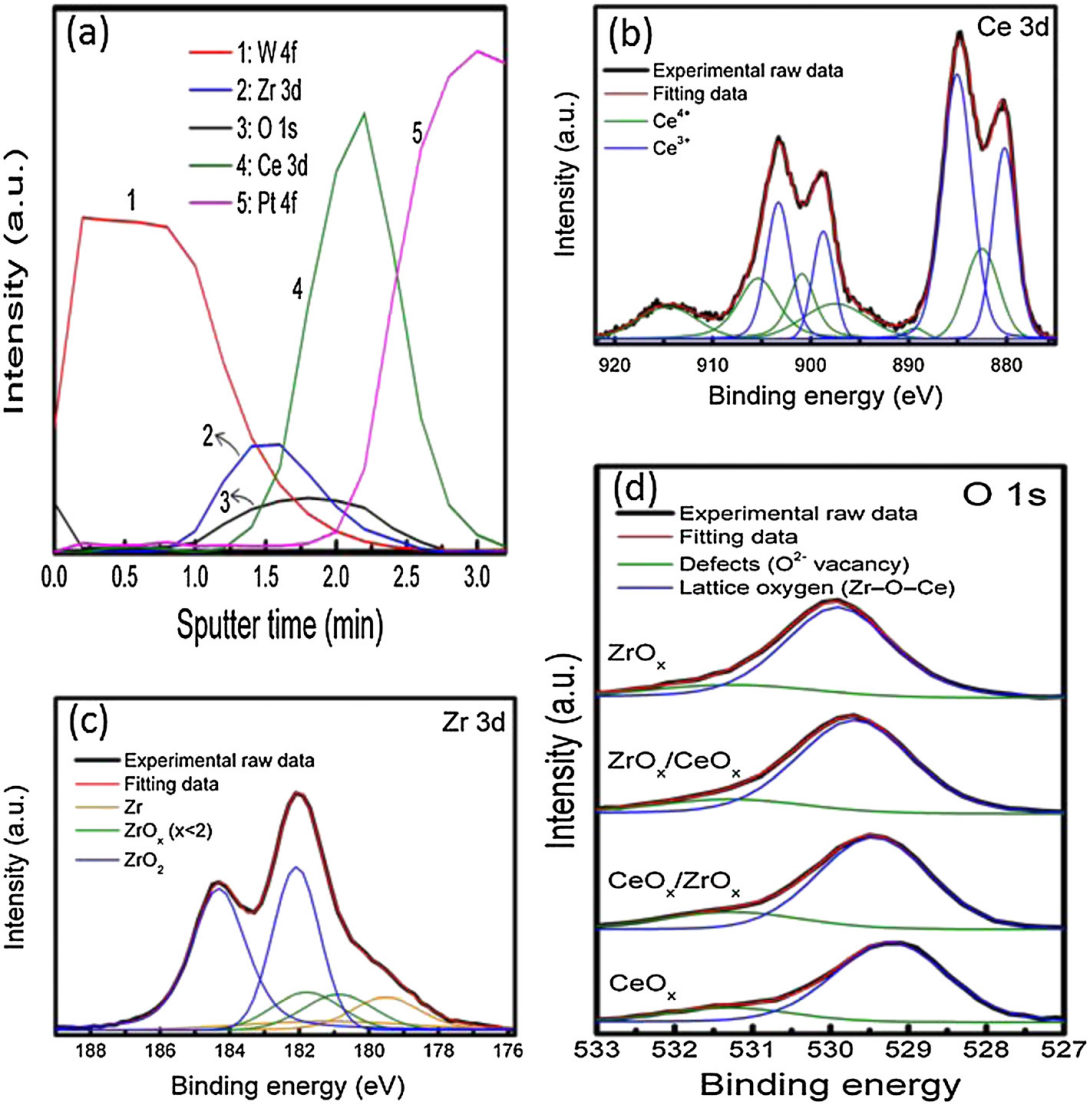
**XPS binding energy profiles. (a)** Depth profiles of Zr, Ce, O, Pt, and W for the W/Zr/CeO_*x*_/Pt structure, **(b)** Ce 3d, **(c)** Zr 3d, and **(d)** O 1 s in the Zr/CeO_*x*_/Pt device.

The ZrO_*y*_ layer is also observed from XPS signals at the interface of Zr and CeO_2_ layers. XPS analysis was carried out to examine the surface chemical composition and the valence/oxidation states of Ce and Zr species involved in the device by inspecting the spectral line shape and signal intensities associated with the core-level electrons. Figure 2a shows the depth profile of chemical composition in the Zr/CeO_*x*_/Pt device. The interdiffusion of O, Ce, and Zr atoms are evident from the spectra. This is an indication of the formation of an interfacial ZrO_*y*_ layer between the CeO_*x*_ and Zr top electrode. The formation of the ZrO_*y*_ layer is further confirmed from the shifting of Zr 3d peaks from a higher binding energy position to lower ones (Figure 2c). The CeO_*x*_ 3d spectrum shown in Figure 2b consists of two sets of spin-orbit multiplets. These multiplets are the characteristics of 3d_3/2_ and 3d_5/2_ (represented as *u* and *v*, respectively) [15]. The spin-orbit splitting is about 18.4 eV. The highest peaks at around 880.2 and 898.7 eV, recognized as *v*_0_ and *u*_0_ respectively, correspond to Ce^3+^ with the highest satellites as *v*′ (885.1 eV) and *u*′ (903.3 eV). Low-intensity peaks, i.e., *v* (882.5 eV) and *u* (900.9 eV) along with satellite features represented as *v*″ (889.4 eV), *v*‴ (897.5 eV), *u*″ (905.4 eV), and *u*‴ (914.6 eV), are observed, corresponding to the Ce^4+^ state.

**Figure 3:**
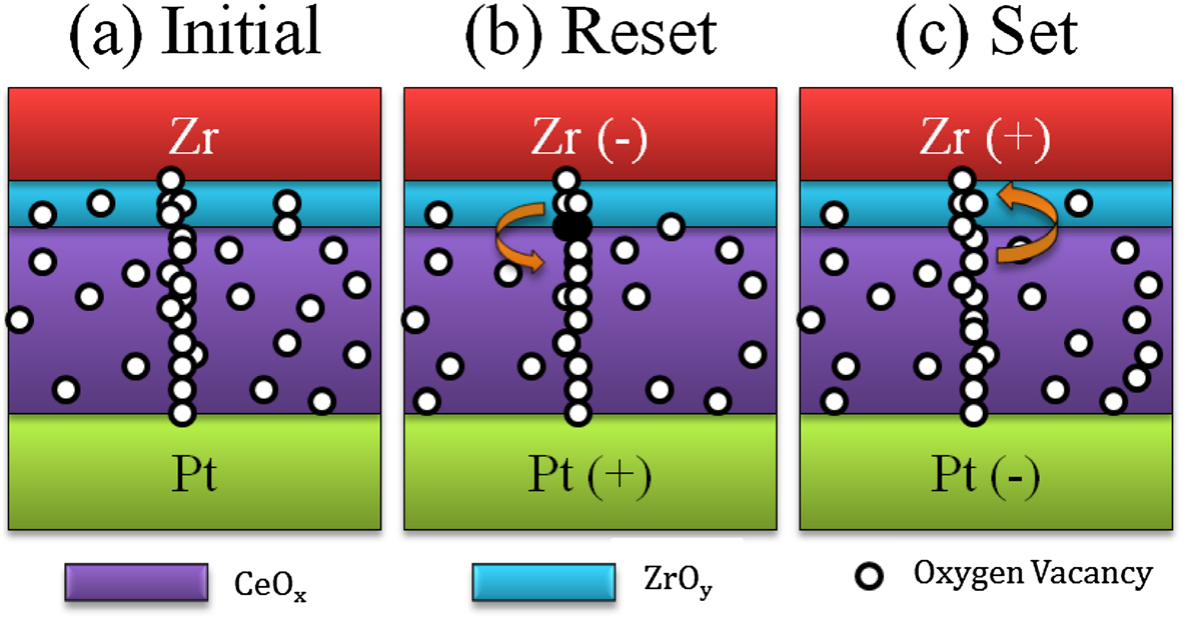
**Schematic of oxygen vacancy-formed multiconducting filaments depicting the switching process in Zr*/*CeO**_***x***_***/*Pt device. (a)** Initial, **(b)** reset, and **(c)** set states. Note that unfilled (filled) circles represent oxygen vacancies (ions) in the CeO_*x*_ films.

In reference to the differentiation between the Ce^3+^ and Ce^4+^ species with different line shapes, the XPS spectra correspond to various final states: Ce(III) = *v*_0_ + *v*′ + *u*_0_ + *u*′ and Ce(IV) = *v* + *v*″ + *v*‴ + *u* + *u*″ + *u*‴ [16]. The presence of the Ce^4+^ state is normally supported by the intensity of the *u*‴ peak, which is known as a fingerprint of Ce(IV) states [16]. This result implies that both Ce^4+^ and Ce^3+^ ions coexist in the bulk as well as in the surface of the CeO_*x*_ film. Concentrations of Ce^4+^ and Ce^3+^, as obtained from the deconvoluted XPS spectra, are 39.6% and 60.4%, respectively. The higher percentage of Ce^3+^ ions indicates that the film is rich of oxygen vacancies [17]. The XPS spectra of Zr 3d exhibit a doublet located at 184.3 and 182.08 eV, as shown in Figure 2c. This doublet corresponds to Zr 3d_3/2_ and Zr 3d_5/2_, respectively [18], as the final states of ZrO_2_. Furthermore, the weak bands at about 181.7 eV assigned to Zr 3d_3/2_ and 180.8 eV assigned to Zr 3d_5/2_ seem to be consistent with the states of ZrO_*y*_ (0 < *y* < 2, 181.6 eV) [19], which also provide an evidence of the formation of a ZrO_*y*_ interfacial layer. Final states of the metallic Zr (3d) are evidenced by the weakest band at 181.2 eV for Zr 3d_3/2_ and 179.5 eV for Zr 3d_5/2_. Figure 2d displays the O 1 s XPS spectra of the Zr/CeO_*x*_/Pt device consisting of peaks at binding energies 529.05, 530.09, and 531.47 eV, which can be attributed to the absorbed oxygen [20], lattice oxygen in CeO_2_[21], and oxygen vacancies [22], respectively. The O 1 s peak is broad due to the nonequivalence of surface O^2–^ ions. In addition to the oxygen vacancies, the preexisting oxygen ions in the Zr/CeO_*x*_/Pt device can also be verified from the spectra. The presence of more than one peak in the O 1 s spectra may have resulted from the overlapping of oxygen from surface defects (the nonlattice oxygen ions), CeO_*x*_, and Zr-O-Ce components as evident from the deconvoluted curves. The deconvoluted peaks detected at 529.2 to 529.9 and 531.47 eV are ascribed to the lattice oxygen and surface defects, respectively. Nonlattice oxygen ions may exist in the grain boundaries and can move with the help of bias voltage. Interaction between the movable oxygen vacancies and oxygen ions in the presence of an external electric field can play an important role in the RS process [23,24]. Based on the above results, a highly stable and forming-free bipolar resistive switching model can be proposed as shown in Figure 3.

**Figure 4:**
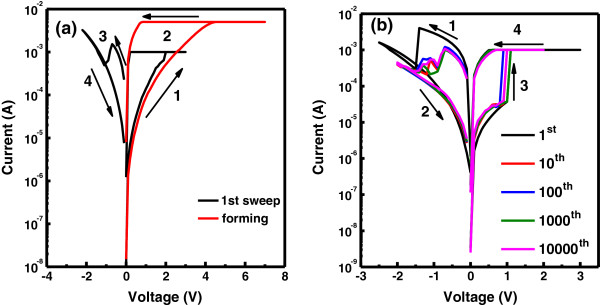
**Typical bipolar (*I*-*V*) curves of resistive switching behavior in Zr/CeO**_***x***_**/Pt devices with different CeO**_***x***_**layer thicknesses. (a)** 25 nm and **(b)** 14 nm.

Figure 4a depicts *I*-*V* bipolar switching characteristics of the Zr/CeO_*x*_/Pt device having a CeO_*x*_ film thickness of 25 nm under DC sweeping at room temperature. Application of positive DC sweeping voltage gradually activates the device, initially forming a conductive path; this process is known as ‘electroforming’ and is similar to defect-induced dielectric soft breakdown. Current gradually increases at the forming voltage (approximately 4 V), and the device is shifted from a high-resistance state (HRS) to a low-resistance state (LRS). At the negative bias of approximately -1.0 V, the current drops abruptly to switch the device from LRS to HRS, known as the reset process. The device returns again to LRS when positive bias exceeds the set voltage (*V*_on_ ~ 2.0 V), and a compliance current of 10 mA is applied to prevent the device from permanent breakdown. The electroforming process usually requires a higher bias and may cause unstable resistance states [8], which make RS characteristics very difficult to modulate. That is why it is valuable to study the resistive switching behavior free from the forming process. In this regard, the thickness of the CeO_*x*_ layer was reduced from 25 to 14 nm in the Zr/CeO_*x*_/Pt devices. It is noticed that by reducing the thickness of the CeO_*x*_ layer, the forming voltage is also reduced. At 14-nm-thick CeO_*x*_, the Zr/CeO_*x*_/Pt device shows a forming-free behavior, as indicated in Figure 4b. Figure 4b shows the first switching cycle of this device. Initially, the device is in LRS [21], so the first reset process (*V*_off_ = -1.4 V) is required to initialize the device by rupturing the preformed conductive filaments between two electrodes, and the device is switched to HRS [22]. A unique resistive switching behavior can be obtained without any forming process, which is more advantageous for the application point of view [2,22]. Conversely, a positive voltage (*V*_on_) of about +1 V is required for the rapid transition of current from HRS to LRS, called the ‘set process.’ During the set process, oxygen vacancies migrate from the top reservoir (ZrO_*y*_ layer) and form conducting filaments [2,4,13,20]. A compliance current of 1 mA was applied to prevent the device from permanent breakdown. An appropriate negative voltage (-0.7 V) is applied to switch the device from LRS back to HRS. During the reset process, the conductive filament is ruptured by the reoxidation of oxygen ions [2,13,22,25].

**Figure 5:**
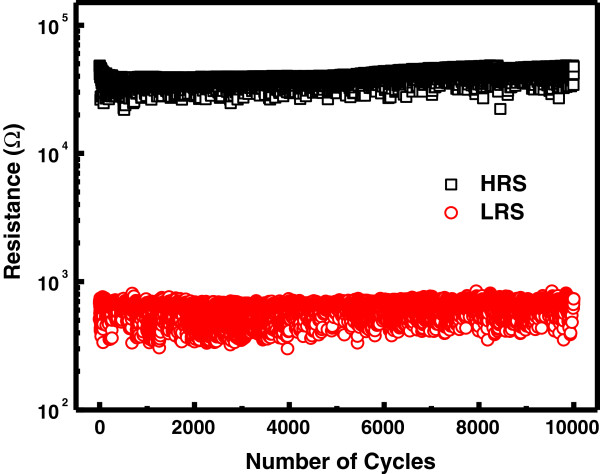
**Endurance characteristic of the Zr/CeO**_***x***_**/Pt device during DC sweeping modes up to 10**^**4 **^**cycles.** The read voltage is 0.3 V.

**Figure 6:**
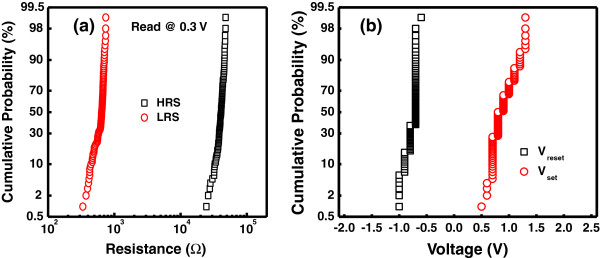
**Statistical and probability distributions. (a)** Statistical distributions of the HRS and LRS measured during switching up to 10^4^ cycles for the Zr/CeO_*x*_/Pt device. **(b)** Probability distributions of *V*_set_ and *V*_reset_.

**Figure 7:**
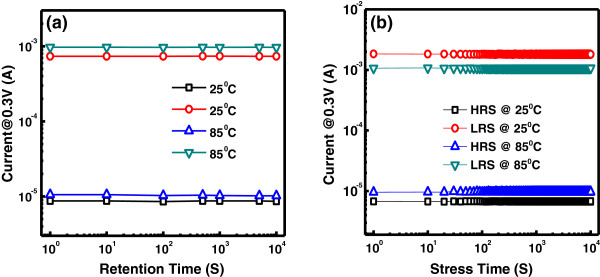
**Retention characteristic and nondestructive readout properties. (a)** Retention characteristic of the Zr/CeO_*x*_/Pt device. The resistance ratios between HRS/LRS are retained for more than 10^4^ s. **(b)** Nondestructive readout properties of both HRS and LRS for 10^4^ s.

To evaluate the memory switching performance of the Zr*/*CeO_*x*_*/*Pt device, endurance characteristics are performed. The memory cell is switched successfully in consecutive 10^4^ switching cycles (*I*-*V* curves) with approximately 40 resistance ratios between HRS and LRS, as shown in Figure 5. Both HRS and LRS are quite stable and no ‘set fail’ phenomena are observed. Figure 6a shows the statistical distribution of LRS and HRS of the device. Furthermore, the device has very good uniformity of resistance values in both HRS and LRS. Figure 6b depicts the distribution of set (*V*_set_) and reset (*V*_reset_) voltages for the device, which shows a narrow range of *V*_reset_ (from -0.5 to -1 V) and *V*_set_ (from 0.5 to 1.3 V) values. The data retention characteristics of the Zr/CeO_*x*_/Pt device are measured at room temperature (RT) and at 85°C, respectively. As shown in Figure 7a, the HRS and LRS are retained stable for more than 10^4^ s at RT and 85°C with a resistance ratio of approximately 10^2^ times at 0.3 V. Hence, suitable read/write durability is obtained. The nondestructive readout property is also verified. As shown in Figure 7b, the two resistance states are stable over 10^4^ s under 0.3 V at RT and 85°C, without any observable degradation.

The RS characteristics of the Zr/CeO_*x*_/Pt device are well explained by the model of filamentary conduction mechanism caused by oxygen ions/vacancies [20,26,27]. Due to impulsive interactions, oxygen vacancies tend to distribute themselves in line patterns and separate from each other in the CeO_*x*_ film [28]. This phenomenon leads to the formation of independent conducting filaments between electrodes instead of their interconnection network. The abundant oxygen vacancies easily form conducting filaments presented in the CeO_*x*_ film, as shown in Figure 3a. The formation mechanism of the conducting filament in the virgin device could be explained as follows: the oxygen vacancies present in the virgin device can be imagined to be formed partially during the deposition of the nonstoichiometric (oxygen deficient) CeO_2_ and partially as a consequence of Zr oxidation. The oxidation of Zr might have increased the concentration of oxygen vacancies in the bulk of the sandwiched nonstoichiometric oxide to such an extent that they formed conductive paths through CeO_*x*_. These conductive filamentary paths composed of oxygen vacancies are somewhat stronger than the filaments that are formed in the subsequent ON states, as indicated by a relatively larger reset power needed for the first reset process (Figure 3b). Such conducting filaments become a cause for the forming-free behavior of the Zr/CeO_*x*_/Pt device. In addition, due to the nonforming process, the current overshoot phenomenon can be suppressed for the following RS [26]. When a negative voltage (*V*_off_) is applied on the top electrode, current flows (i.e., the electrons injected from the top electrode) through the conductive filaments that produce local heating at the interface along with the repelled oxygen ions from the ZrO_*y*_ layer, causing local oxidization of the filaments at the interface between ZrO_*y*_ and CeO_*x*_ layers. This oxidization causes the rupture of filaments and the switching of the device to HRS [29], as shown in Figure 3b. Figure 3c depicts the set process; the device can switch from HRS to LRS by applying a positive bias voltage on the Zr top electrode, which causes the drift of oxygen vacancies from the ZrO_*y*_ interfacial layer down to CeO_*x*_ and the oxygen ions simultaneously upward. The conducting filament consisting of oxygen vacancies is formed. In this RS model, the ZrO_*y*_ interfacial layer behaved as an oxygen reservoir in the device. Besides being an oxygen reservoir, the ZrO_*y*_ interfacial layer also acts as an ion barrier [30], which may lead to the good endurance property of the Zr/CeO_*x*_/Pt structure.

**Figure 8:**
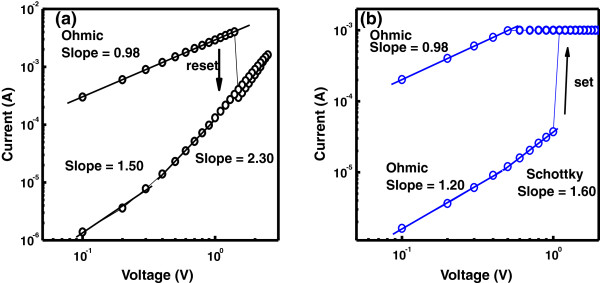
***I*-*V *curves of the Zr/CeO**_***x***_**/Pt memory device are displayed in double-logarithmic scale.** The linear fitting results in both ON state and OFF state: **(a)** positive-voltage region and **(b)** negative-voltage region. The corresponding slopes for different portions are also shown.

In order to elucidate the conduction mechanisms of the device, the *I*-*V* curve is plotted in the double-logarithmic mode, both the positive and negative bias regions, as shown in Figure 8a,b, respectively. The conduction mechanism being responsible for charge transport in the low-voltage region involves ohmic behavior (since *n* = 1), but it is different in the medium- and high-voltage regions for the device, where the conduction behavior can be well described by the space charge-limited current (SCLC) theory [31-36]. Ohmic conduction in LRS is assumed to be caused by the oxygen vacancies which probably provide shallow energy levels below the conduction band edge. The SCLC mechanism is generally observed when the electrode contacts are highly carrier injecting. Due to the formation of an interfacial ZrO_*y*_ layer between Zr and CeO_*x*_ films, the conduction mechanism in the device behaves according to the SCLC theory since the ZrO_*y*_ layer is known to provide electron trapping sites and to control the conductivity by trapping and detrapping.

## Conclusions

Resistive switching characteristics of the Zr/CeO_*x*_/Pt memory device were demonstrated at room temperature. The conduction mechanisms for low- and high-resistance states are revealed by ohmic behavior and trap-controlled space charge-limited current, respectively. Oxygen vacancies presented in the CeO_*x*_ film and an interfacial ZrO_*y*_ layer was formed, as confirmed by XPS and EDX studies. Long retention (>10^4^ s) at 85°C and good endurance with a memory window of HRS/LRS ≥ 40 were observed. This device has high potential for nonvolatile memory applications.

## Competing interests

The authors declare that they have no competing interests.

## Authors’ contributions

The manuscript was written through the contributions of all authors, MI, CYH, DP, CJH, TLT, JHJ, CAL, UC, AMR, EA, IT, MYN, and TYT. All authors read and approved the final manuscript.
